# Supplementary material for: Effects of sprint interval training compared to high intensity interval training on repeated sprint capacity and sport-specific performance in college-aged male tennis players
Source: PLoS One. 2025 Sep 19;20(9):e0332705. doi: 10.1371/journal.pone.0332705 (PMC12449013; doi:10.1371/journal.pone.0332705)
Supplement: Attachment — Submitted filename: comments.docx [file pone.0332705.s003.docx]

Dear Editor and Reviewers,

We sincerely thank you for the time and effort you have invested in reviewing our manuscript entitled “Effects of sprint interval training compared to high intensity interval training on repeated sprint capacity and sport-specific performance in college-aged male tennis players.” We highly appreciate the constructive and detailed comments, which have significantly improved the quality and clarity of our work.

We have carefully addressed all the concerns raised by both reviewers and have revised the manuscript accordingly. A detailed point-by-point response has been prepared and is included below, with specific modifications made in the manuscript highlighted accordingly.

Reviewer #1: General Comments

The manuscript investigates the effects of sprint interval training (SIT) compared to high-intensity interval training (HIIT) on tennis-specific performance in collegiate male tennis players. This research addresses a relevant topic in sports science, as optimizing training protocols for tennis players can enhance performance and potentially reduce injury risk.

Major Weaknesses:

1. Intervention Duration Inconsistency: The study describes both a "six-week" (line 107) and "8-week" intervention (lines 19, 233), creating confusion about the actual duration of the study.

Thank you for pointing out this inconsistency. We have reviewed the manuscript and have now unified the intervention duration throughout the study. All references to the intervention period have been changed to **8 weeks** to eliminate any confusion and ensure consistency. The relevant sections have been updated accordingly.

2. Methodological Clarity Issues: The SIT and HIIT protocols lack precise descriptions of progression throughout the intervention period. Additionally, the manuscript suggests that training was conducted "during the off-season" (line 117) but also "during the preparatory season" (line 90), which is contradictory.
Thank you for your valuable feedback. To address the methodological clarity issues:

We have now provided additional details on the progression of the SIT and HIIT protocols. Specifically, we clarified how the intensity, volume, and recovery periods were gradually adjusted during the 8-week intervention to ensure a progressive overload. These changes have been incorporated in the **Training Program** section, with specific details on how the intensity was gradually increased week by week.

Regarding the inconsistent reference to the training period, we apologize for the confusion. We have revised the manuscript to clarify that the intervention took place during the **preparatory season**, not the off-season. The term “off-season” was incorrectly used in some parts of the manuscript, and we have now updated the text to reflect the correct training phase. All references to the timing of the intervention have been standardized to **preparatory season**.

We hope these revisions address your concerns and improve the clarity of our methods.

3. Statistical Reporting Inconsistencies: Some statistical results are reported with varying formats and levels of detail throughout the manuscript. The reporting of effect sizes is inconsistent, and some p-values do not match across text and tables.

We sincerely thank the reviewer for this insightful comment. In response, we have carefully revised the statistical reporting throughout the manuscript to ensure consistency and clarity. S

4. Lack of Blinding Procedures: There is no mention of any blinding procedures for testers during pre- and post-intervention assessments, which could have introduced bias.

Thank you for your valuable comment. We apologize for the oversight in not explicitly describing the blinding procedures. In fact, the assessors who conducted the pre- and post-intervention performance tests were blinded to group allocation to minimize potential measurement bias.

5. Limited Discussion of Physiological Mechanisms: While the authors attempt to explain the physiological mechanisms behind the observed differences between SIT and HIIT, these explanations lack sufficient references to supporting literature.
Thank you for this insightful comment. In response, we have expanded the Discussion section to provide a more detailed explanation of the physiological mechanisms underlying the superior effects of SIT over HIIT, particularly in terms of repeated sprint ability, aerobic capacity, and tennis-specific performance. We have also incorporated additional references to support our interpretations, including literature on neuromuscular adaptations, metabolic responses, and central/peripheral fatigue mechanisms. The revised content can be found in the second and third paragraphs of the Discussion section.

Minor Weaknesses:

1. Several grammatical and typographical errors throughout the manuscript.
Thank you for your valuable comment. In response, we have carefully proofread the entire manuscript and corrected all identified grammatical and typographical errors. We have ensured that sentence structures are clear and concise, and that academic language is used consistently throughout the paper. Additionally, the revised manuscript has been reviewed by a native English speaker with expertise in academic writing to further improve language accuracy and readability. We hope that the current version meets the journal’s standards.

2. Figures lack clear legends and comprehensive captions.
Thank you for your helpful suggestion. We have revised all figure legends and captions to ensure they are clear, comprehensive, and self-explanatory.
3. Incomplete discussion of limitations and generalizability of findings.
Thank you for this valuable comment. We have revised the final paragraph of the Discussion section to provide a more comprehensive reflection on the study’s limitations and the generalizability of our findings. Specifically, we acknowledged the lack of blinding for participants, the relatively small and homogeneous sample (male adolescent tennis players), and the short duration of intervention. We also discussed how these factors may limit the extrapolation of results to other populations (e.g., females, elite athletes, older adults) and settings. The revised paragraph is now included at the end of the Discussion section.
4. Table 3 presents inconsistent formatting of statistical significance markers.
Thank you for your observation. We have carefully reviewed and revised Table 3 to ensure that all statistical significance markers are formatted consistently.
5. Limited discussion of practical applications for tennis coaches and practitioners.
Thank you for highlighting this important point. In response, we have added a new paragraph at the end of the Discussion section to better articulate the practical implications of our findings for tennis coaches and practitioners. This includes specific guidance on how sprint interval training (SIT) can be integrated into training routines to enhance both aerobic capacity and on-court performance in youth tennis players. We believe this addition enhances the real-world relevance and applicability of our research
Specific Comments

Line 18-19: The study is described as involving 30 tennis players for an "8-week intervention," but later in line 107, it's referred to as a "six-week court-based SIT intervention." Clarify the actual duration of the intervention.
Thank you for identifying this inconsistency. We confirm that the intervention lasted for **eight weeks** for both groups. The reference to a “six-week court-based SIT intervention” in line 107 was incorrect and has now been revised to reflect the actual eight-week duration. We have carefully checked the manuscript to ensure that all references to the intervention period are now consistent.

Line 90-91: The study claims recruitment was from "January 20, 2025, to January 25, 2025" which is a future date. Please correct.
Thank you for pointing this out. We confirm that participant recruitment occurred between January 20 and January 25, 2025. The entire intervention and post-assessment were completed shortly thereafter, and the manuscript was prepared and submitted following the completion of data collection and analysis. We have double-checked the timeline to ensure all dates are chronologically consistent and clarified where necessary to avoid confusion.

Line 90-95: The manuscript states the study was conducted "during the preparatory season," but later (line 117) mentions it was "during the off-season." Clarify which is correct.
Thank you for highlighting this inconsistency. The study was conducted during the **preparatory phase of the annual training cycle**, which directly followed the off-season and preceded the competitive season. To avoid confusion, we have revised the terminology throughout the manuscript and now consistently refer to this period as the “preparatory season.”

Line 108: The phrase "a longitudinal and randomized controlled experimental design" is redundant since randomized controlled already implies experimental design.
Thank you for pointing this out. We agree that the phrase was redundant. We have revised the sentence to improve clarity and avoid unnecessary repetition.

Line 111: More details on the stratified block randomization procedure are needed, including what factors were used for stratification.
Thank you for your valuable comment. We have revised the manuscript to provide more details on the stratified block randomization procedure.

Line 135-136: The Hit and Turn Test description refers to "Figure 1," but this figure appears to be the CONSORT flow diagram, not the test setup. This creates confusion.
Thank you for pointing out this inconsistency. We have corrected the figure reference in the description of the Hit and Turn Test to refer to the correct figure that depicts the test setup. The CONSORT flow diagram remains as Figure 1, and the illustration of the Hit and Turn Test has been updated as Figure 2. All corresponding figure citations in the text have been adjusted accordingly to avoid confusion.

Thank you for your careful observation. We have revised the figure and accompanying description to ensure alignment. The figure now clearly illustrates the 6 × 20 m shuttle sprints with turning points and timing gates, as described in the text. The textual description has also been revised for consistency with the updated figure layout.

Line 149-154: The RSA test description references "Figure 2," but the description doesn't fully match what is shown in the figure. Improve the clarity of the figure or description.
Thank you for your suggestion. We have clarified the description of the RSA test to better match the illustration shown in Figure 3. The text now explicitly details the shuttle sprint pattern (5 m + 10 m + 5 m) and its relevance to tennis-specific movement, and the figure has been enhanced with directional arrows and annotations to reflect this sequence more clearly.

Line 184-190: The description of the TEST protocol lacks clarity on how ball accuracy was determined. Provide specific criteria.
Thank you for pointing this out. We have revised the protocol description in Lines 184–190 to provide detailed criteria for determining ball accuracy, including the dimensions and location of the accuracy target zone, the classification rule for accurate hits, and how the accuracy rate was calculated.

Line 198-205: The methods for measuring ball velocity and accuracy need more detailed description, particularly the validity and reliability of the PlaySight system for these measurements.
Thank you for your valuable comment. In the revised manuscript, we have added a more detailed description of how ball velocity and accuracy were measured using the PlaySight SmartCourt system. Specifically, we clarified that the system uses multiple high-speed cameras and computer vision algorithms to track shuttlecock trajectories and landing locations in real-time. To further address your concern regarding the reliability of the PlaySight system in our specific research context, we conducted a test–retest analysis involving 10 randomly selected participants who repeated the same protocol within a 7-day interval under identical testing conditions. Intraclass correlation coefficients (ICC) were calculated using a two-way random-effects model (absolute agreement), yielding values of 0.96 for ball velocity and 0.92 for ball accuracy.

Line 229-230: The statement that "The training program took place during the preseason period" contradicts earlier mentions of "preparatory season" and "off-season."
Thank you for your careful observation. To ensure consistency throughout the manuscript, we have revised the wording in Line 229–230 and standardized the terminology to “preparatory period,” which corresponds to the off-season phase before formal competitions. This correction ensures alignment with earlier descriptions and removes potential confusion.

Line 251-262: More details are needed on how TRIMP was calculated and used to ensure equal training loads between groups.
Thank you for your insightful comment. We have revised this section to provide a detailed explanation of how TRIMP was calculated using the Edwards' method. We also described how training load was continuously monitored and adjusted to ensure weekly TRIMP equivalence between groups within an acceptable range (±10%).

Line 271: The statement about "outliers (defined as studentized residuals exceeding 3 standard deviations from zero) were identified and removed" should specify how many outliers were removed and from which variables.
Thank you for pointing this out. We have revised the manuscript to clarify that outliers were screened using studentized residuals (±3 SD), but no data points met the criteria for exclusion. Therefore, no outliers were removed from any variable.
Line 279-284: The results reporting for maximal completed level shows inconsistent formatting of statistics compared to other results.
Thank you for your careful review. We have revised the reporting format for the maximal completed level results to ensure consistency with other reported outcomes throughout the manuscript.

Line 310-312: The paper reports that "Blood lactate concentration showed significant reduction only in HIIT group" but doesn't explain why this might have occurred or its practical significance.
Thank you for your valuable comment. We have revised the discussion section to include a detailed explanation of this finding.

Line 339-346: This paragraph makes claims about SIT's effectiveness on repeated sprint capacity without sufficient citations to support the mechanisms proposed.

*Thanks. We have added the citations to support the mechanisms proposed.*

Line 371-390: The explanation of physiological mechanisms lacks sufficient references, particularly when discussing muscle fiber recruitment, lactate clearance, and autonomic regulation.

*Thank you. We have added the references about the muscle fiber recruitment, lactate clearance, and autonomic regulation.*

Line 392-399: The limitations section fails to discuss important limitations such as lack of a control group, potential learning effects in the testing protocols, and the homogeneous sample.

*Thanks for your comments. We have rewritten the limitations sections.*

Lines 414-506: The manuscript would benefit from incorporating more recent and relevant literature related to sport-specific testing, training load quantification, and interval training methodologies. I recommend the authors consider including the following references to strengthen their literature framework and discussion of mechanisms:
1. Dhahbi, W., Hachana, Y., Souaifi, M., Souidi, S., & Attia, A. (2024). Tennis-specific incremental aerobic test (TSIAT): construct validity, inter session reliability and sensitivity. Tunisian Journal of Sports Science and Medicine, 2(1), 25-32. This paper provides validated tennis-specific testing protocols that would enhance your methodological approach and interpretation of sports-specific adaptations.
2. Dhahbi, W., Chaabene, H., Pyne, D. B., & Chamari, K. (2024). Standardizing the quantification of external load across different training modalities: a critical need in sport-science research. International Journal of Sports Physiology and Performance, 19(11), 1173-1175. This work addresses the critical issue of training load standardization across different modalities like SIT and HIIT, which would strengthen your methodology section and discussion of comparative effects.
3. Turki, O., Dhahbi, W., Padulo, J., Khalifa, R., Ridène, S., Alamri, K., ... & Chamari, K. (2019). Warm-up with dynamic stretching: positive effects on match-measured change of direction performance in young elite volleyball players. International Journal of Sports Physiology and Performance, 15(4), 528-533. This research examines performance enhancement in an intermittent sport with change-of-direction demands similar to tennis, offering parallels to your study's assessment of repeated sprint ability.
4. Farhani, Z., Ghouili, H., Dhahbi, W., Ammar, A., Ben Aissa, M., Bouzouraa, M. M., ... & Ben Ezzeddine, L. (2025). Optimizing the number of players and training bout durations in soccer small‐sided games: Effects on mood balance and technical performance. European Journal of Sport Science, 25(3), e12208. This recent work explores technical performance preservation during high-intensity intermittent exercise, which aligns with your findings on technical stability during tennis-specific performance.
5. Dhahbi, W., Briki, W., Heissel, A., Schega, L., Dergaa, I., Guelmami, N., et al. (2025). Physical Activity to Counter Age-Related Cognitive Decline: Benefits of Aerobic, Resistance, and Combined Training - A Narrative Review. Sports Medicine (Open), In press. This forthcoming review discusses the differential physiological adaptations to various training modalities including combined approaches, which would enhance your discussion of the mechanisms underlying the superior effects of SIT observed in your study. Incorporating these recent references would strengthen the theoretical framework, methodological justification, and interpretation of your findings, particularly in relation to sport-specific adaptations and the physiological mechanisms underlying the observed training effects.

*Thank you. We have used these references to strengthen their literature framework and discussion of mechanisms:*

Table 3: The formatting of the table is inconsistent, with some cells containing multiple decimal places while others have different precision. Standardize the presentation.
Thank you for pointing this out. We have revised Table 3 to ensure consistency in numerical presentation.
Figure 4: The figure showing pre-post changes is difficult to interpret without better labeling and a more detailed caption explaining the statistical significance markers.

Thank you for your insightful comment. We have revised Figure 4 to enhance clarity and interpretability.

Reviewer #2: Title: Effects of sprint interval training compared to high intensity interval training on repeated sprint capacity and sport-specific performance in college-aged male tennis players
This study compared the effects of sprint interval training (SIT) and high-intensity interval training (HIIT) on repeated sprint capacity and tennis-specific performance in male college tennis players. After an 8-week intervention, the SIT group showed more significant improvements in tennis-specific performance (ball accuracy, tennis performance index), repeated sprint ability (RSATT, RSAbest, Sdec), and aerobic capacity (VO₂max) compared to the HIIT group. Although both training methods increased aerobic fitness, it was concluded that SIT is a more efficient strategy for high-intensity intermittent sports such as tennis.
Thank you for the interesting insights your article provides. The manuscript is well-written and deals with an important topic. I have thoroughly reviewed the manuscript and would like to make some suggestions for your review.

"Line 30: The abbreviations 'OBLA' and 'VT2' should be written in their long forms at first use, followed by the abbreviations in parentheses. For example: 'the onset of blood lactate accumulation (OBLA) and second ventilatory threshold (VT2)'."
Thank you for your helpful suggestion. We have revised the sentence on Line 30 to define the abbreviations at first use.
Line 88: The sample size is small (n = 15 per group), and no power analysis is provided to support the adequacy of the design for detecting meaningful effects.
Thank you for raising this important point. While our sample size was constrained by the availability of competitive collegiate tennis players, we conducted a post-hoc power analysis using G*Power 3.1.

Line 111: selection of subjects
The manuscript mentions "stratified block randomization" but fails to specify:
Stratification criteria (e.g., baseline VO₂max, tennis experience, or performance metrics).
Block size (e.g., 4 or 6 participants per block) and allocation ratio (e.g., 1:1 for SIT vs. HIIT).
Method of sequence generation (computerized tool? manual randomization?).
Without these details, the reproducibility of the study is compromised. please add the required explanation
Thank you for your careful observation. We have revised the Methods section to clarify the randomization procedure in detail.

Line 230: "Further clarification on the standardization of training protocols is needed.
Load Intensity Comparison: Although HIIT and SIT protocols used different work-to-rest ratios, the total workload (e.g., mechanical load) and metabolic stress (e.g., EPOC) were not matched between conditions. This makes it unclear whether the observed adaptations resulted from the training type itself or from unequal load distribution.
Thank you for this insightful comment. We have clarified the degree of training standardization in the revised manuscript. In addition to the clarifications provided in the Methods section, we have now explicitly acknowledged the lack of precise matching in external load and metabolic stress as a study limitation. This has been added to the final paragraph of the Discussion section to enhance transparency and interpretability of our findings.

Line 238: While the HIIT protocol used tennis-specific movements, the SIT used general sprints. This leaves it unclear whether SIT's superiority in technical skills is due to training specificity or a genuine physiological advantage. This section requires further clarification.

Thank you for this insightful comment. We agree that the difference in task specificity between the SIT and HIIT protocols may confound the interpretation of technical performance outcomes. Although SIT lacked tennis-specific movement patterns, it led to greater improvements in technical skills, possibly due to the superior physiological adaptations it elicited.

Line 327: Enhance the discussion section using references.

- Morais, J. E., Kilit, B., Arslan, E., Bragada, J. A., Soylu, Y., & Marinho, D. A. (2024). Effects of On-Court Tennis Training Combined with HIIT versus RST on Aerobic Capacity, Speed, Agility, Jumping Ability, and Internal Loads in Young Tennis Players. Journal of Human Kinetics, 95, 173.

- Fernandez-Fernandez, J., Sanz-Rivas, D., Kovacs, M. S., & Moya, M. (2015). In-season effect of a combined repeated sprint and explosive strength training program on elite junior tennis players. The Journal of Strength & Conditioning Research, 29(2), 351-357.

- Brechbuhl, C., Schmitt, L., Millet, G. P., & Brocherie, F. (2018). Shock microcycle of repeated-sprint training in hypoxia and tennis performance: Case study in a rookie professional player. International Journal of Sports Science & Coaching, 13(5), 723-728.

*Thank you. We have used these references to enhance the discussion section.*

Table 3: should list the abbreviated terms in the first column, with their full explanations provided at the end of the table.
Thank you for your helpful suggestion. We have revised Table 3 by retaining the existing format of the main outcome variables and added a dedicated list of abbreviations below the table, where each term is defined in full. This revision enhances the clarity of the table while maintaining consistency with the presentation of statistical data. The updated Table 3 now includes a clearly formatted abbreviations section that explains all shortened terms used in the table.

Line 414: the reference section needs to be checked
References 1 and 35 are the same
References 19-22 are the same
References 19-28 needs to be checked

Thank you for your careful review and for pointing out the issues in the reference section.
