# Supplementary material for: Effects of sprint interval training compared to high intensity interval training on repeated sprint capacity and sport-specific performance in college-aged male tennis players
Source: PLoS One. 2025 Sep 19;20(9):e0332705. doi: 10.1371/journal.pone.0332705 (PMC12449013; doi:10.1371/journal.pone.0332705)
Supplement: Attachment — Submitted filename: comments0825.docx [file pone.0332705.s004.docx]

We would like to sincerely thank the editor and reviewers for their thorough evaluation of our manuscript and for the constructive feedback provided. Your insightful comments have greatly helped us to refine and strengthen the methodological and statistical aspects of our study. In response to your assessments, we provide the following clarifications and revisions to further enhance the transparency and rigor of our work.

Reviewer #1: General Comments

The authors have made substantial improvements to the manuscript following the previous review. The study design comparing sprint interval training (SIT) to high-intensity interval training (HIIT) in tennis players addresses an important research question with practical applications. The methodology is generally sound, and the statistical analyses are appropriate. However, several minor issues require attention before the manuscript can be accepted for publication.

Specific Comments

Abstract: Page 1, Line 19: The phrase "8-week intervention" should be clarified as "8-week training intervention" to specify the nature of the intervention more precisely.

*Thank you for your valuable suggestion. We have revised the phrase “8-week intervention” to “8-week training intervention” in the Abstract to specify the nature of the intervention more precisely.*

Page 2, Line 25: The statistical reporting format is inconsistent. Consider standardizing the presentation of F-values, p-values, and effect sizes throughout the abstract.

*Thank you for your helpful comment. We have carefully revised the Abstract to standardize the reporting format of statistical results.*

**Introduction: Page 3, Line 65: The sentence beginning "This suggests that both HIIT and SIT might be beneficial..." creates an awkward transition. Consider restructuring this paragraph for better flow.**

*Thank you for this insightful comment. We agree that the transition in this paragraph was abrupt and could disrupt the flow of ideas. In response, we have restructured the paragraph to improve logical coherence and ensure a smoother progression of arguments. Specifically, we clarified the connection between the physiological demands of tennis and the relevance of both HIIT and SIT by integrating the supporting literature in a more cohesive manner. The revised paragraph now highlights the complementary nature of HIIT and SIT in tennis conditioning, providing a stronger rationale for our study focus. Please see the revised paragraph in the Introduction section.*

**Page 4, Line 85-88: The hypothesis statement could be more specific about the expected magnitude of differences between training modalities.**

*Thank you for your suggestion. In response, we have revised the hypothesis statement to clearly specify the expected differential effects of SIT and HIIT. The updated version reflects our anticipation that SIT would produce larger improvements in repeated sprint performance, sport-specific skills, and aerobic endurance, while both protocols were expected to yield similar benefits in general physiological measures such as VO₂max and lactate threshold. Please see the revised hypothesis at the end of the Introduction section*

Methods: Page 5, Line 92-93: The statement "Recruitment and data collection were completed between January and March 2025, prior to manuscript preparation" contains a future date that appears to be an error. This needs immediate correction.

Page 5, Line 99: Similarly, "The study subjects' recruitment period was from January 20, 2025, to January 25, 2025" uses future dates that require correction.

*Thank you for pointing out this issue. We confirm that the study was indeed conducted in 2025 and had been fully completed before manuscript preparation. To avoid confusion, we have revised the statements in the Methods section to read: “The study subjects were recruited between January 20 and January 25, 2025, and all data collection was completed prior to manuscript preparation.”*

Page 6, Line 131: The phrase "conducted during the preparatory season" appears twice in close proximity. Consider removing the redundancy.

*Thank you for noting this redundancy. We have revised the Methods section (Page 6, Line 131) by removing the repeated phrase “conducted during the preparatory season” and rephrasing the sentence for conciseness.*

Page 8, Line 176: The calculation formula for Sdec should be presented more clearly, perhaps as a separate equation rather than embedded in the text.

*Thank you for the helpful suggestion. We have revised the Methods section to present the calculation formula for Sdec as a separate equation rather than embedding it within the text.*

Results: Page 15, Line 319: The F-value presentation format should be consistent throughout. Some instances use F(1,28) while others omit the degrees of freedom format.

*Thank you for your valuable comment. We have revised the Results section to ensure that all F-values are consistently presented in the standard format F(df1, df2) = value (e.g., F(1, 28) = 22.649).*

Page 16, Line 343: The phrase "435，50±19.73*" contains what appears to be a formatting error with the comma placement.

*Thank you for identifying this formatting error. We have corrected “435，50±19.73*” to “435.50 ± 19.73*” in the Results section to ensure accuracy and consistency in numerical.*

**Discussion: Page 18, Line 375: The sentence "These effects are likely mediated by SIT-induced physiological adaptations..." could benefit from more specific mechanistic details.**

*Thank you for your valuable feedback. We appreciate the suggestion to provide more mechanistic specificity regarding the physiological adaptations induced by sprint interval training (SIT). In response, we have revised the relevant paragraph in the Discussion section to elaborate on the specific neuromuscular and metabolic mechanisms underlying the observed performance improvements. The updated text now describes (1) increased recruitment of type II muscle fibers contributing to explosive power and sprint performance, (2) enhanced mitochondrial biogenesis and oxidative enzyme activity improving energy efficiency and recovery, and (3) upregulation of monocarboxylate transporters (MCT1 and MCT4) facilitating improved lactate clearance. These mechanisms are now explicitly linked to our key performance outcomes (e.g., RSAbest, RSATT, Sdec, and [La]max), as shown below:*

*“These effects are likely mediated by a range of SIT-induced physiological adaptations. First, the maximal sprint nature of SIT promotes greater recruitment of type II muscle fibers, which are crucial for explosive efforts such as repeated sprinting and directional changes seen in tennis. This neuromuscular adaptation enhances the rate of force development, contributing to improvements in RSAbest and RSATT. Second, SIT enhances mitochondrial biogenesis and increases oxidative enzyme activity (e.g., citrate synthase, succinate dehydrogenase), which support improved aerobic energy production during recovery phases between sprints. Third, SIT has been shown to upregulate monocarboxylate transporters (MCT1 and MCT4), facilitating more efficient lactate transport and clearance, thereby delaying fatigue as evidenced by the reduced Sdec and lower [La]max observed in our study. Together, these adaptations enable athletes to maintain sprint performance across repeated efforts while preserving technical execution during prolonged match-like conditions.”*

*We hope this revision addresses the reviewer’s concern and enhances the clarity and depth of the mechanistic explanation.*

**Page 21, Line 461: The practical recommendations section would benefit from more specific implementation guidelines for coaches.**

*Thank you for this helpful suggestion. In response, we have revised the practical recommendations section to include more specific and actionable implementation guidelines for coaches. The updated text now provides example SIT and HIIT protocols, including sprint distances, recovery intervals, session frequency, and timing within the training cycle. We also offer guidance on how coaches can tailor these protocols based on individual athlete needs, training phases, and internal load monitoring (e.g., RPE, HRV). These additions aim to enhance the practical utility of our findings for sport practitioners. The revised section reads as follows:*

*“From a practical standpoint, incorporating SIT sessions 2–3 times per week during the preparatory phase may enhance repeated sprint ability and tennis-specific performance. A typical SIT protocol can involve 6–8 all-out sprints over 20–30 meters, with 20–30 seconds of passive recovery. Sessions may be scheduled after technical drills or on separate days, depending on training goals. Coaches can adjust sprint volume, recovery duration, and session frequency based on the athlete’s fitness level, training phase, and recovery capacity.*

*For general aerobic conditioning, HIIT protocols (e.g., 4 × 4 min at 90–95% HRmax with 3-min recovery) may be used earlier in the training cycle. A periodized approach—using HIIT in early phases and SIT closer to competition—can help maximize both endurance and sport-specific performance. Monitoring internal load through RPE or heart rate can help ensure appropriate adaptation and recovery.”*

*We hope this revision provides greater clarity and value for coaching professionals and meets the expectations of the reviewer.*

Tables and Figures: Table 3, Page 33: The formatting of statistical significance markers could be more consistent. Some entries use asterisks while others use different notation systems.

*Thank you for your suggestion. We have revised Figure 3 to ensure that the statistical significance markers are presented in a consistent format.*

Figure 4, Page 35: The figure caption could be more descriptive about what specific changes are being highlighted.

*Thank you for your helpful suggestion. We have revised the caption of Figure 4 to provide a more descriptive explanation of the specific changes highlighted.*

References: Page 24-31: Several references contain formatting inconsistencies, particularly in journal name abbreviations and page number presentations.

*Thank you for pointing out these issues. We have carefully revised the References section (Pages 24–31) to ensure consistency in formatting.*

Statistical and Methodological Assessment

The statistical approach using repeated-measures ANOVA is appropriate for the study design. The post-hoc power analysis addresses concerns about sample size adequacy. Effect size reporting enhances the interpretation of practical significance. The randomization procedure appears sound, though more details about allocation concealment would strengthen the methodology section.

*We sincerely appreciate the reviewer’s positive assessment of our statistical approach, post-hoc power analysis, and effect size reporting. We acknowledge the reviewer’s suggestion regarding allocation concealment. In the revised manuscript, we have added clarifications on the randomization process. Specifically, randomization was performed using a computer-generated random sequence, and group assignments were carried out by an independent researcher who was not involved in recruitment or assessment. This ensured that allocation concealment was maintained throughout participant assignment. These details have been incorporated into the Methods section to strengthen the transparency and rigor of the study methodology.*
